# Notoamide R: A Prominent Diketopiperazine Fermentation Metabolite amongst Others of *Aspergillus ochraceus* in the Absence of Ochratoxins

**DOI:** 10.3390/molecules28083518

**Published:** 2023-04-17

**Authors:** Peter Mantle, Andrew Roberts, Claire Beaumont

**Affiliations:** 1 Biochemistry Department and Centre for Environmental Policy, Imperial College London, London SW7 2AZ, UK; 2 Analytical Development, GlaxoSmithKline, Stevenage SG1 2NY, UK; 3 Drug Metabolism and Pharmacokinetics, GlaxoSmithKline, Stevenage SG1 2NY, UK

**Keywords:** notoamide R, biosynthesis, ochratoxins, toxicology, diketopiperazine, radiolabel

## Abstract

Ochratoxin A is historically the most notable secondary metabolite of *Aspergillus ochraceus* on account of its toxicity to animals and fish. Currently, over 150 compounds of diverse structure and biosynthesis is a challenge to predict the array for any particular isolate. A brief focus 30 years ago on the failure to produce ochratoxins in foods in Europe and the USA revealed consistent failures to produce ochratoxin A by isolates from some USA beans. Analysis for familiar or novel metabolites particularly focused on a compound for which mass and NMR analyses were inconclusive. Resort to ^14^C-labelled biosynthetic precursors, particularly phenylalanine, to search for any close alternative to ochratoxins, was combined with conventional shredded-wheat/shaken-flask fermentation. This yielded, for an extract, an autoradiograph of a preparative silica gel chromatogram, which was subsequently analysed for an excised fraction using spectroscopic methodologies. Circumstances then delayed progress for many years until the present collaboration revealed notoamide R. Meanwhile, pharmaceutical discovery around the turn of the millennium revealed stephacidins and notoamides, biosynthetically combining indole, isoprenyl and diketopiperazine components. Later, in Japan, notoamide R was added as a metabolite of an *Aspergillus* sp. isolated from a marine mussel, and the compound was recovered from 1800 Petri dish fermentations. Renewed attention to our former studies in England has since shown for the first time that notoamide R can be a prominent metabolite of *A. ochraceus*, sourced from a single shredded wheat flask culture with its structure confirmed by spectroscopic data, and in the absence of ochratoxins. Renewed attention to the archived autoradiographed chromatogram allowed further exploration, but in particular has stimulated a fundamental biosynthetic approach to considering influences redirecting intermediary metabolism to secondary metabolite accumulation.

## 1. Introduction

The discovery of ochratoxin A (OTA) about sixty years ago [1] in South Africa as a metabolite of *Aspergillus ochraceus* Wilh. was related to evidence of general toxicity to experimental rats. It was soon also recognised as a metabolite of a *Penicillium* mould associated with seasonal losses in the Danish bacon industry [2]. The use of local cereals, incompletely dried and stored during damp harvest conditions predisposing them to unrecognised moulding, could prove toxic. The specific target was pig kidneys, associated with poor carcass weight and rejection by meat inspection. Further toxicological evidence demonstrated rat kidney cancer, mainly in males, in response to subclinical gavage dosing with OTA within a period of 2 years [3]. OTA then became a popular putative cause of idiopathic Balkan endemic nephropathy [4] and was declared as a possible risk for human cancer [5]. Investigations into the possible health risks of tropical sources have naturally focused on *A. ochraceus.* Notably, surveys of food spoilage fungi in Balkan nephropathy villages in Croatia and Bulgaria found little trace of *A*. *ochraceus* [6,7]. However, inclusion in the Bulgarian survey of two UK grocery samples of USA beans plainly revealed *A. ochraceus*, even after surface sterilisation, with its identity verified by the Commonwealth Mycological Institute. However, tests for the biosynthesis of ochratoxins failed for all isolates [7]. Later, an exploratory submerged fermentation in London of one of the USA isolates in potato dextrose broth (Difco) confirmed the absence of OTA, normally recognised easily in TLC by its blue fluorescence under UV_350_ light. Instead, a prominent non-fluorescent metabolite occurred, identifiable under UV_254_ light. Electron impact MS revealed a putative M^+^ 429, while ^1^H and ^13^C NMR data eluded interpretation.

For the present report, subsequent complementary investigation employed renewed spectroscopic analysis at GlaxoSmithKline to reveal notoamide R. Additionally, the exploration of chromatographic fractions of the radioactive biosynthetic precursor experiment, based on previous experience [8], offers further steps towards understanding the biosynthetic consequences of failing to form ochratoxins.

## 2. Results

### 2.1. Notoamide R

**Figure 1:**
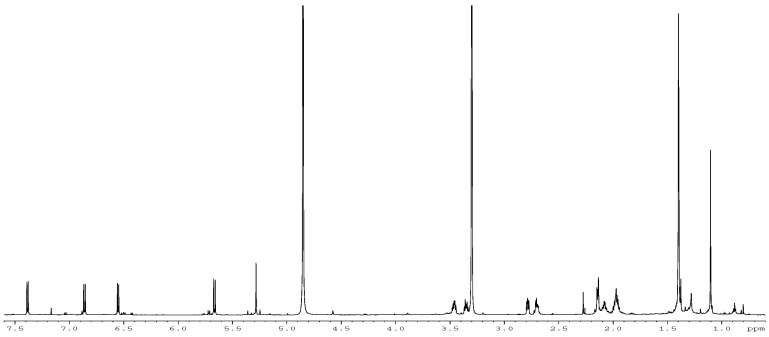

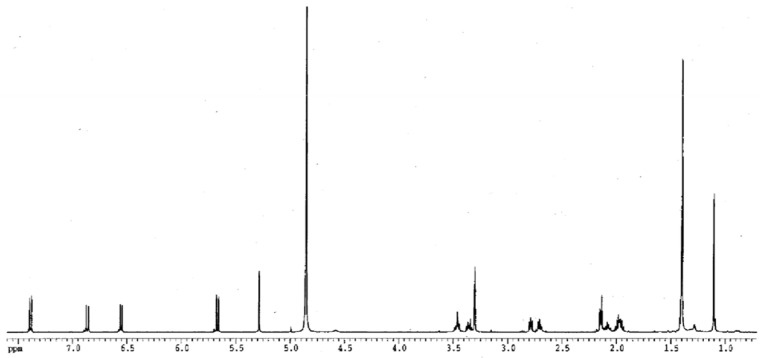
Comparative ^1^H NMR spectrum for current Notoamide R, above, and literature data, below.

**Figure 2:**
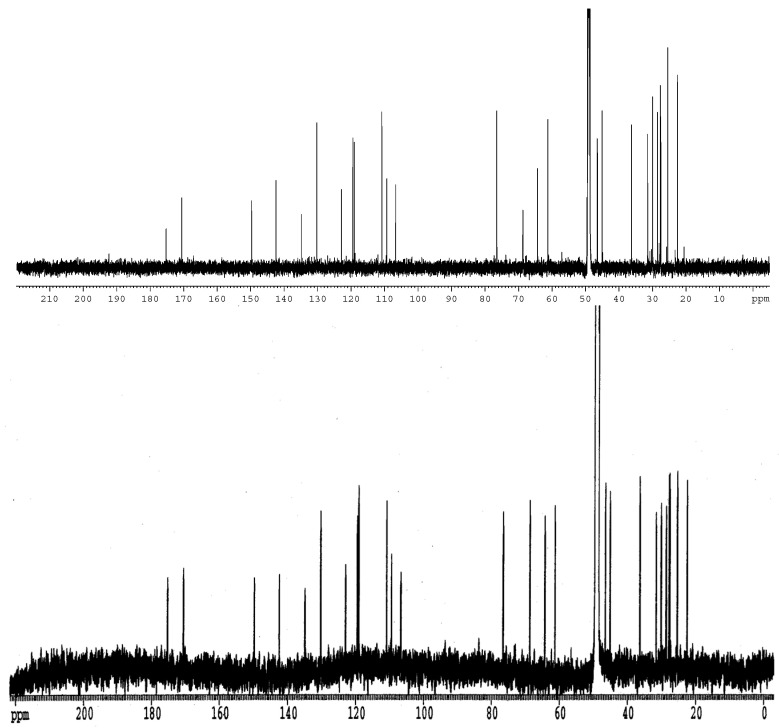
Comparative ^13^C NMR spectrum for current Notoamide R, above, and literature data, below.

**Figure 3:**
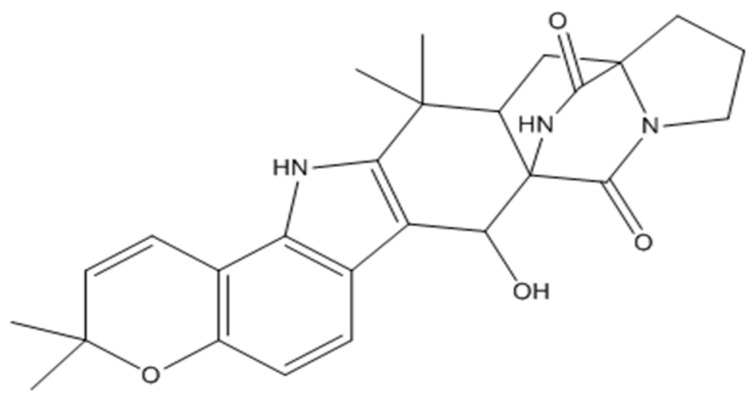
The chemical structure of Notoamide R.

Renewed analysis of the isolated metabolite, formerly with a mass of 429, revealed the ion as a fragment of a molecular ion (M + H) 448.2248 matching a molecular formula of C_26_ H_30_N_3_ O_4_ relating to 21-OH stephacidin. The former mass had thus simply corresponded to a loss of water. ^1^H, ^13^C, HSQC, COSY, ROESY, ^13^C-HMBC and ^15^N-HMBC NMR data were collected from the sample. Preliminary analysis of the NMR data was able to readily identify the structure of the first three unsaturated rings of the sample which indicated that the unknown metabolite likely belonged to the Notoamide/Stephacidin class of molecules. Before any further spectrosopic analysis was performed on the sample, a literature search was carried out to compare the available literature data with that acquired here. An excellent match was made with the ^1^H and ^13^C NMR data (Figure 1 and Figure 2), including that previously obtained on Notoamide R [9], confirming the structural identity of the isolated component as in Figure 3. NMR spectra were recorded on a Bruker Avance 700 MHz NMR spectrometer in methanol-d_4_. Chemical shifts were referenced to the residual solvent peaks (δ_H_ 3.30 ppm and δ_C_ 49.0 ppm). Mass spectra were recorded on a Waters Q-Tof Premier with electrospray ionisation.

### 2.2. Biosynthesis

**Figure 4:**
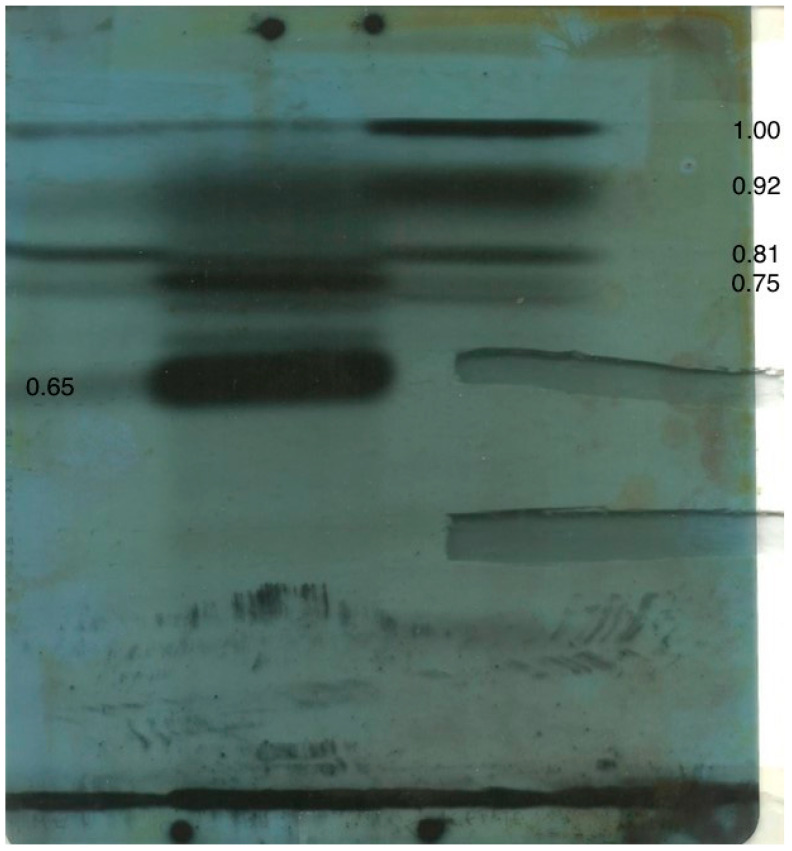
Preparative chromatogram resolving isolated extrolites from four shaken culture fermentations of an *Aspergillus ochraceus* isolate not producing ochratoxins, each fed with a radiolabelled amino acid. Additionally overlaid is the autoradiograph revealing corresponding apparent biosynthetic incorporations of radiolabelled probes. Tracks (L→R) for methionine, phenylalanine, acetate, glycine. To aid discussion in this text, the top limit of radioactivity in the chromatogram, deduced from cellular sterol, is designated as Rf 1.0. The image also shows two silica gel bands excised for extrolite elution and structural analysis; the dark edges are an artefact of scanning.

The chromatography and subsequent autoradiography results of extracts of an *A. ochracous* fermentation fed with either methionine, phenylalanine, acetate or glycine, as described in the Methods and Materials, are illustrated in Figure 4. Details will be discussed subsequently, but the component tracks for the four test radiolabels are clearly illustrated and present several prospective matches. Notably, glycine did not contribute in the main display but contributed with all others to radioactivity across the origin, representing unresolved compounds in the particular chromatography solvent, chosen as the diagnostic in case ochratoxins were present. Viewing the chromatogram under UV_254_ light revealed two regions with potential for further identification; at about Rf 0.65, there is an unlikely but exact correlation with the prominent ^14^C-phenylalanine derivative.

## 3. Discussion

A recent comprehensive review of the secondary metabolites of *A. ochraceus* [10] is a key source for the present exploration of combinations of metabolites where the principal animal toxin (OTA) is not synthesised. This valuable review illustrates 165 extrolites attributed to isolates of this species categorised biosynthetically as, for example, isocoumarins, polyketides, indole alkaloids and diketopiperazines according to structure; this structure relates to biosynthetic routes and sources. Eventually, a compilation of the compound compositions of biotypes or ecotypes of *A. ochraceus* would be helpful, and the present study works towards defining the secondary metabolism in the absence of perhaps the most well-known product in this area, ochratoxins. Refinement of the present radiolabelling approach could be a step towards this. However, experimentally, for the notoamide R identified in the first wave of recent investigation here, application in the subsequent radiochemical approach (Figure 4) to the displayed metabolites is difficult. This is inevitable because a different chromatography solvent was applied to the fermentation products. No indication of isoprene involvement, via acetate, is evident at Rf 0.65 in Figure 4. It is recognised that a second addition of ^14^C-acetate as a putative precursor could have confirmed that conclusion by fully satisfying the cell membrane demand for sterols (Rf 1.0) during mycelial growth. Nevertheless, several notable combinations for acetate-derived compounds are evident at and above Rf 0.75, with at least three combinations with a methylation from methionine. Some of the above clues to extrolite identity appear linked to all or parts of the U-^14^C-phenylalanine explored, but the acetate/methionine pair at Rf 0.81 could fit diaporthin/orthosporin, listed as isocoumarins for *A. ochraceus* [10]. The biosynthesis of these isocoumarins has already been explored for these two precursors [11].

Apparent radioactivity from phenylalanine at around Rf 0.65 presents a challenge for interpretation, but its close adjacency to evidence of methylation is quite compelling. Since the excision of the UV_254_-absorbing zone in the Rf 0.65 region had been accurate, it is possible to see an imperfect fit between the excised region and the radioactivity pattern from phenylalanine and know that it is just the evidence of that experimental precursor that requires interpretation. The future of phenylalanine and its perfectly correlated methyl donation can thus be disassociated from involvement in any notoamide. It remains to be discovered if there is any substance linked to the prominent phenylalanine-derived radiolabel. Theoretically, this aromatic amino acid might easily have been transformed into ^14^C-tyrosine. Phenylalanine catabolism to chorismate within the aromatic pathway seems to have not been recognised in intermediary metabolism, although enzymic flexibility might have evolved within the global distribution of fungi conforming to the *A. ochraceus* description. Otherwise, redirection to indolic metabolites via tryptophan might then be an option for the present dilemma.

A key difference between the biosynthesis of ochratoxins and notoamides concerns access to the branched primary metabolic pathway to aromatic amino acids for the end products phenylalanine and tryptophan. Attention to any reversibility of enzymology in the phenylalanine branch might point to variable secondary metabolism within *A. ochraceus* by redirecting that pathway to notoamides, as an alternative to ochratoxins.

Shaken natural substrate fermentation for a high yield of a secondary metabolite such as OTA was particularly useful not only during biosynthetic studies [12], but also for exploiting yield potential for animal toxicology studies [13,14]. Its application across sixteen *A. ochraceus* isolates from Asia, Africa, South America and Australia [15] listed cases with a trace of OTA, but four gave at least 1 mg/g of original substrate. The Australian isolate was subsequently used extensively to create experimental renal tumours in male rats from lifetime dietary exposure [13,14]. A Brazilian form from the 2000 survey [15] excelled for producing radiolabelled OTA of high specific activity [16]. This was revealed in comparative studies to select the optimal exploitation of the commencement of ochratoxin pentaketide component synthesis relative to demand on acetate for primary metabolism.

The fungal production of diketopiperazine alkaloids was extended by the recognition of stephacidins A and B around the turn of the millennium [9,17], variously attributed to an *Aspergillus* sp., and both indole and isoprenyl components were recognised. Subsequently, notoamides were defined as an extension of stephacidin biosynthesis and notoamide R was described as a product of an *A. ochraceus* isolated from a Japanese marine mussel [18].

The perceived involvement of proline in the notoamide R diketopiperazine moiety here, its shared role in the unique amino acid purpurolic acid of *Claviceps purpurea* [19], and its implied involvement in highly focal rat renal apoptosis by metabolites of *Penicillium polonicum* [20] are notable. These studies highlight proline’s unique cyclic potential to influence the unusual structural conformation of some fungal metabolites to express interesting pharmacological properties that have been independently observed [21]. Several notoamides have shown interesting potential concerning L-Dopa deficiency in Parkinson’s disease [22], but no human pharmacological potential seems to have yet been explored for notoamide R. The cerebral biosynthesis of L-Dopa from the tyrosine exogenously synthesised by plants and fungi is vital for efficient brain function. Coincidentally, L-Dopa is also a key compound in the formation of marine adhesive proteins, such as those found in mussels from which the original notoamide R-producing *Aspergillus* was isolated [23].

The fermentation protocol used here for enabling the secondary metabolite revelation of new idiolytes in a somewhat atypical fungus, via putative ^14^C-labelled biosynthetic precursors, is the same in principle as that applied to the toxicology of OTA in rats [24]. It reported on a single dose of ^14^C-OTA to male rats, but failed to detect significant DNA adducts in kidneys or livers. Notably, there was no mention of the source or construction of ^14^C-OTA. An expanded version appeared a year later [25]. In 2005, the same authors illustrated Figure 1 in [26] on the completed urinary elimination of OTalpha (the polyketide part of OTA) two days after ceasing five days of OTA dosing to rats. That elimination was replicated through a subsequent similar dosing cycle. To have delayed, as reported in 2003 and 2004, kidney and liver analysis for DNA adducts until three days after giving only one gavage dose of the special ^14^C-OTA (production subsequently fully illustrated [16]) in an unspecified volume of corn oil would have guaranteed a negative finding. The radiolabel in the OTA was only in the OTalpha component. Maximum blood plasma concentration of OTA from a 5 ppm OTA daily diet takes about a month to reach about 8 µg/mL for an adult rat [27]. Ideally, this should guide the design of any test for OTA/DNA adducts. A detailed description of the experimental production of the special ^14^C-OTA (0.25 mCi/mmol) from ^14^C-sodium acetate (>100 mCi/mmol supplied from Hartmann Analytic, Braunschweig, Germany) has since been produced [16]. Thus, the claim to have discredited the finding of male rat renal DNA adducts [28] is scientifically invalid. The ^14^C-OTalpha (the standard excretory product of OTA provided biosynthetically by the acetate-derived pentaketide) from only a small dose from the original 50 mg of ^14^C-OTA would all have been excreted in rat urine over the course of three days. The waste of the special ^14^C enrichment is regrettable but, if that ^14^C-OTA still exists, the experiment should be repeated and properly designed to ensure that added OTA can be circulating in kidney parenchyma at the time of analysis. About 3 months of continuous rat ingestion of OTA is necessary to cause any renal cancer [27]. Several days of the ^14^C-OTA treatment should be used experimentally to explore adducts of DNA. Therefore, the 2006 EFSA statement [29] was questionable and the more recent update [30] that ‘The possible formation of specific OTA-induced DNA adducts remains highly controversial’ could be easily resolved.

Meanwhile, an extensive genome survey within the wide diversity of the yellow-spored *Aspergillus* spp., coupled with the corresponding cultural illustration on standard media [31], has made correlations concerning three extrolite types: OTA; penicillic acid; and the napthoquinones xanthomegnin, viomellein and vioxanthin. All of these are designated as metabolites of *A. ochraceus* and have a wide range of occurrence in other fungal genera. The present approach to exploring biosynthetic explanations of apparent inconsistencies of OTA occurrence in *A. ochraceus* thus has considerable fundamental value.

*A. ochraceus* has long been the original source of one of the first potent mycotoxins, and even in the South African context some isolates apparently did not produce ochratoxins in laboratory culture. The search for its convincing threat to human renal health, specifically in the form of the Balkan Endemic Nephropathy, was not convincing [6,7] and even a random test on some American beans failed to reveal OTA as a metabolite of this fungus. The subsequent listing of many extrolites [10] did not reveal new potent toxins. However, complex pharmaceutical exploration revealed that stephacidins and notoamides expressed therapeutic potential for Parkinson’s disease by augmenting Levodopa medication [32]. Whether the biosynthesis of some of these metabolites of *A. ochraceus* only occurs when OTA biosynthesis fails is now an open question stimulated by the present study. However, it is based on the fundamental aromatic amino acid biosynthetic pathway.

## 4. Methods and Materials

Shaken solid substrate fermentation was carried out [9] using 500 mL Erlenmeyer flasks containing 40 g autoclaved shredded wheat breakfast cereal (Cereal Partners, Welwyn Garden City, Hertfordshire, England). Sterile water (16 mL) was added and the flask was shaken to mix the moistened substrate. The freely sporing inoculum of an *A. ochraceus* isolate from surface-sterilised red kidney beans from the USA had been shown experimentally to not produce ochratoxin A [7]. The isolate, grown on a similar substrate, was added (1 g) and the flask was incubated at 25 °C stationary for 3 days to proliferate biomass and sporulation before transference to a rotary shaker (200 rpm) with a 10 cm eccentric throw. Preliminary fermentation development had shown that OTA production had commenced within 3 days, at which stage ^14^C-labelled biosynthetic precursors could be added for the indicative demonstration of involvement in the secondary metabolism. For the present search for the production of phenylalanine derivatives other than ochratoxins, 5 µCi of L-[^14^C (U)]-phenylalanine was added to one flask. Similarly, to illustrate any polyketides, [1-^14^C]-acetic acid sodium salt was added to another to recognise common cellular sterols. L-[methyl-^14^C]-methionine was included to recognise methyl group donation. L-[^14^C (U)]-glycine was added to another, partly as a control being involved rather rarely in fungal secondary metabolism. Shaken fermentation continued at 25 °C for a further 4 days before cultures were extracted with ethyl acetate, as is usual for OTA. After evaporation to small volume, extracts were applied across the origin of a preparative (2 mm thick) silica gel chromatography plate (Camlab, Cambridge, UK). The silica gel contained a fluorescent dye which, on irradiation with UV_254_ light, could reveal absorbing compounds. The chromatogram was developed with toluene/ethyl acetate/formic acid (5:4:1). No coloured or fluorescent (UV_350_) components were evident, confirming the absence of ochratoxins. Autoradiography (Kodak X-ray film) for 2 days revealed a contrasting incorporation of putative precursors.

## Data Availability

Not applicable.

## Sample Availability

The chromatogram in Figure 4 has been archived, and a reference sample of notoamide R is available, for further explorations.
